# To control and to be controlled: understanding the *Arabidopsis* SLIM1 function in sulfur deficiency through comprehensive investigation of the EIL protein family

**DOI:** 10.3389/fpls.2014.00575

**Published:** 2014-10-22

**Authors:** Anna Wawrzyńska, Agnieszka Sirko

**Affiliations:** Institute of Biochemistry and Biophysics, Polish Academy of SciencesWarsaw, Poland

**Keywords:** transcription factor, EIL protein family, EIN3, SLIM1, *Arabidopsis*, sulfur, ethylene

## Abstract

Sulfur limitation 1 (SLIM1), a member of the EIN3-like (EIL) family of transcription factors in *Arabidopsis*, is the regulator of many sulfur deficiency responsive genes. Among the five other proteins of the family, three regulate ethylene (ET) responses and two have unassigned functions. Contrary to the well-defined ET signaling, the pathway leading from sensing sulfate status to the activation of its acquisition *via* SLIM1 is completely unknown. SLIM1 binds to the 20 nt-long specific UPE-box sequence; however, it also recognizes the shorter TEIL sequence, unique for the whole EIL family. SLIM1 takes part in the upregulation and downregulation of various sulfur metabolism genes, but also it controls the degradation of glucosinolates under sulfur deficient conditions. Besides facilitating the increased flux through the sulfate assimilation pathway, SLIM1 induces microRNA395, specifically targeting ATP sulfurylases and a low-affinity sulfate transporter, SULTR2;1, thus affecting sulfate translocation to the shoot. Here, we briefly review the identification, structural characteristics, and molecular function of SLIM1 from the perspective of the whole EIL protein family.

## INTRODUCTION

Sulfur is present in various compounds due to its ability to readily change the oxidation state. The majority of sulfur in living organisms is in the reduced form of organic sulfur and thiols, while the environment offers predominantly oxidized inorganic sulfate. Only plants (and algae) together with fungi and bacteria are capable of sulfate assimilation and its reduction, therefore playing a pivotal role in the biogeochemical sulfur cycle. The availability of sulfur in the soil fluctuates, therefore plants constantly have to adapt to the changing environment by reprogramming their metabolism. Modulation of gene expression at the level of transcription is a major control point in multiple biological processes, thus the main interest of many researchers was to identify transcriptional regulators specific for sulfur deficiency signaling. Sulfur limitation 1 (SLIM1) from *Arabidopsis thaliana*, so far the only described transcription factor strictly assigned to this pathway, was found in an elegant genetic approach exploiting the fluorescent sulfur deficiency responsive reporter (Maruyama-Nakashita et al., 2006). It has to be stressed out, however, that SLIM1 regulates only a set of genes of sulfur metabolism and also other factors are controlling the gene expression during sulfur limitation.

## THE EIL FAMILY OF TRANSCRIPTIONAL REGULATORS

Sulfur limitation 1 was previously identified as the gene *ETHYLENE-INSENSITIVE-LIKE 3* (*EIL3*) coding for a putative transcription factor of unknown function (Guo and Ecker, 2004).

It belongs to a small family of proteins found exclusively in plants of which several members have been cloned and characterized across various species, including *Arabidopsis* (Chao et al., 1997), tobacco (Kosugi and Ohashi, 2000; Rieu et al., 2003), tomato (Tieman et al., 2001), maize (Gallie and Young, 2004), carnation (Iordachescu and Verlinden, 2005), rice (Mao et al., 2006), kiwi (Yin et al., 2010), and cucumber (Bie et al., 2013). In the *Arabidopsis* genome, there are six genes annotated to encode the EIL family proteins [ethylene-insensitive3 (EIN3) and EIL1–EIL5; Guo and Ecker, 2004]. EIN3 together with its functional homologues EIL1 and EIL2 are transcription factors controlling the expression of ethylene (ET)-responsive genes (Chao et al., 1997; Solano et al., 1998). EIL3/SLIM1 seems to be a specific regulator of sulfur deficiency response since only SLIM1 from the EIL family complemented the phenotype of the *slim1* mutants (Maruyama-Nakashita et al., 2006). Additional proof that SLIM1 mediated regulation is separated from the ET response pathway is that the set of SLIM1-dependent genes are not regulated by the ET precursor 1-aminocyclopropane 1-carboxylic acid (Maruyama-Nakashita et al., 2006). The roles of EIL4 and EIL5 in plant metabolism so far have not been defined (Guo and Ecker, 2004). The first cloned gene of the family, *EIN3*, was identified through positional cloning in the collection of ET-insensitive *Arabidopsis* mutants (Chao et al., 1997). The family is characterized by highly acidic N-terminal amino acids, five small clusters of basic amino acids scattered mostly in the first half of the protein and a proline-rich domain (Chao et al., 1997). The EIL family proteins are highly homologous to one another mainly in their N-terminal half of around 300 amino acid residues. Sequence-specific DNA-binding activities of EIN3, EIL1, EIL2 proteins have been demonstrated using electro-mobility shift assay (Solano et al., 1998; Kosugi and Ohashi, 2000). The location of the unique DNA-binding domain in the primary structure of an EIL protein was identified based on the SLIM1 sequence using the surface plasmon resonance technique (Yamasaki et al., 2005). The structure consists of five alpha-helices, packing together into a globular shape as a whole, possessing a novel fold dissimilar to known DNA-binding domain structures.

## ETHYLENE SIGNALING PATHWAY

**FIGURE 1:**
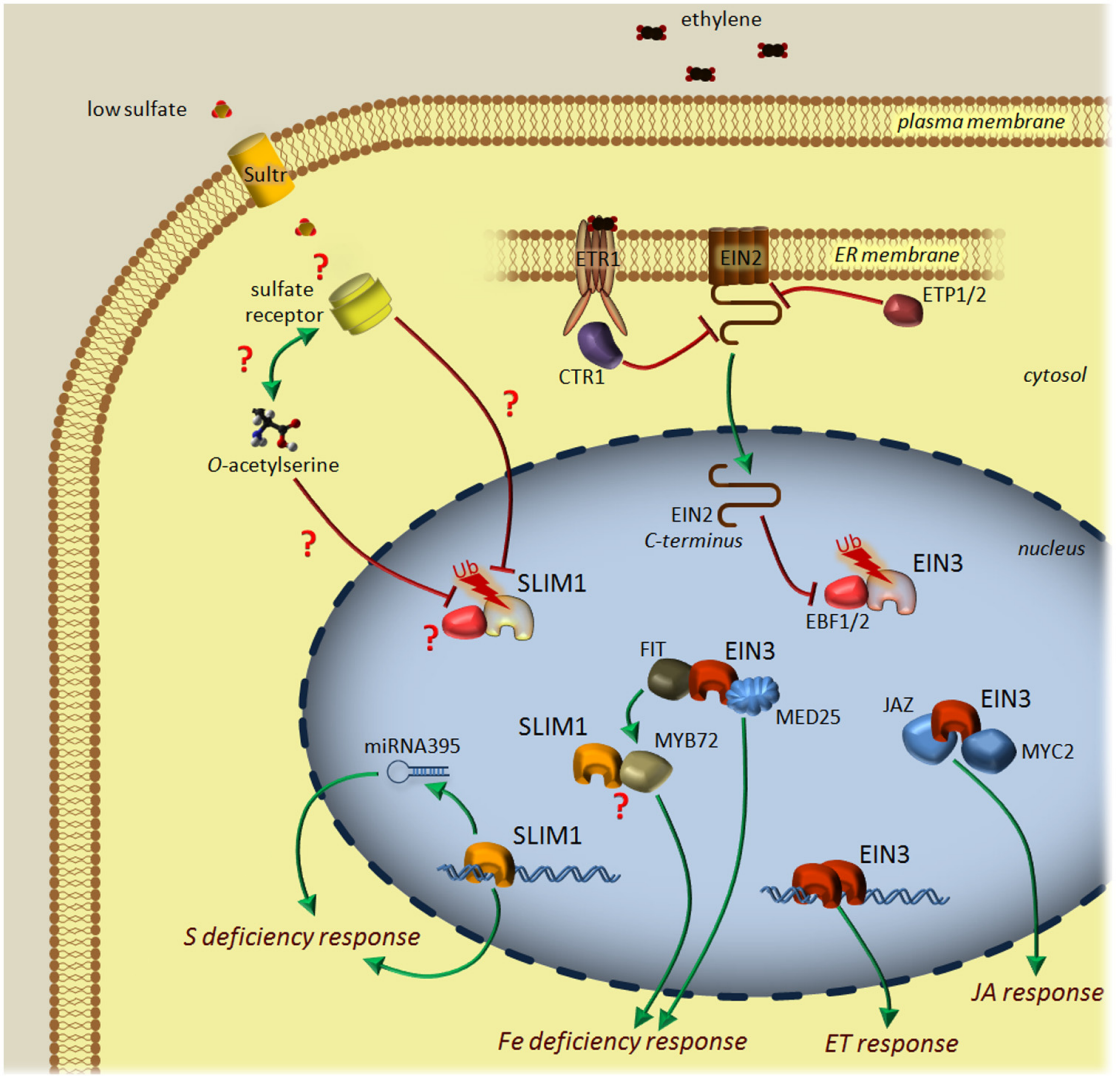
**Current model of the ethylene (ET) and sulfur deficiency signaling pathways in *Arabidopsis*.** In contrast to ET signaling, sulfur deficiency signaling is poorly characterized. Sulfate is transported to cytosol *via* sulfate transporters of the Sultr family. Low sulfate availability is sensed by an unknown receptor and may depend on *O*-acetylserine level. The low sulfur (LSU) signal is transmitted to the nucleus and putatively stabilizes transcriptional factor SLIM1. SLIM1 induces the transcription of selected genes and miRNA395, thus reprogramming the transcriptional profile to answer the sulfur deficiency conditions. ET is perceived by the receptor proteins (for example, ETR1) present in the ER membrane. When the hormone is absent, the receptors activate a Ser/Thr kinase, CTR1, that dimerizes and suppresses the ET response by inactivating EIN2 through the phosphorylation of its C-terminal end. The EIN2 protein level is negatively regulated by the F-box proteins ETP1 and ETP2 and proteasomal degradation, while two other F-box proteins, EBF1/2 serve for the degradation of the transcription factor EIN3 in the nucleus to shut off the ET response. Upon perception of ET, ETR1 inactivates CTR1 and promotes the cleaving off of the C-terminal end of EIN2 that induces the degradation of EBF1/2 after import to the nucleus. EIN3 dimerizes and activates a transcriptional cascade of ET-responsive genes. Depending on the other environmental factors, EIN3 also interacts with JAZ proteins and transcriptional factor MYC2 to shape the jasmonic acid (JA) response. Another partner of EIN3 is MED25, which is a part of a complex regulating iron homeostasis. Additionally, EIN3 binds to FIT, a central regulator of iron deficiency response affecting the transcription level of many genes, with MYB72 among them. MYB72 can interact with SLIM1; however, the outcome of this interaction is unknown. Positive (green) and negative (red) lines represent activation and downregulation processes, respectively. SLIM1 and EIN3, shown in fading colors with Ub (ubiquitin), correspond to proteins marked for proteasome-mediated degradation. Question marks depict the points that are still waiting to be addressed by researchers.

The best characterized protein of the *Arabidopsis* EIL family is EIN3, which together with EIL1, mediates most, if not all, plant responses to ET. The gaseous phytohormone ET regulates many aspects of the plant life cycle, including seed germination, root hair development, root nodulation, flower senescence, leaf abscission, and fruit ripening (Johnson and Ecker, 1998). The emission of ET is tightly controlled by internal signals during development as well as environmental stimuli, including nutritional deficiencies. An initially linear pathway of ET signaling was drawn using a number of molecular genetic studies (Guo and Ecker, 2004). However, latest research presents a much more complex pathway with multiple feedback loops and control levels (see Merchante et al., 2013 for review). A family of five endoplasmic reticulum-associated receptors perceives ET. There are two types of ET receptors in *Arabidopsis*. ETR1 and ERS1 contain three transmembrane domains and a conserved histidine kinase domain, and have been shown to form homodimers. ETR2, EIN4, and ERS2 have four membrane-spanning regions and a degenerate histidine kinase domain that lacks one or more elements necessary for catalytic activity. A copper cofactor, which is delivered by the copper transporter responsive to antagonist-1 (RAN1), is required for ET binding (Wang et al., 2002). In the absence of an ET signal, receptors activate a Ser/Thr kinase, CTR1, that dimerizes and suppresses the ET response (**Figure 1**). ET binding leads to the functional inactivation of receptors and the disability of CTR1 to phosphorylate a positive component of the pathway – the membrane located EIN2. The non-phosphorylated C-terminal end of EIN2 is cleaved off by an unknown mechanism and is transferred to the nucleus (Merchante et al., 2013). The level of EIN2 is regulated by the F-box proteins ETP1 and ETP2, and its degradation via the 26S proteasome. Two other F-box proteins, EBF1 and EBF2, control the level of transcription factors EIN3/EIL1 in the nucleus, thus shutting off the transcription of the ET response genes in the absence of the signal (**Figure 1**). Upon perception of ET, the C-terminal end of EIN2 stabilizes EIN3/EIL1 and induces degradation of EBF1 and EBF2. Additionally, the levels of mRNAs encoding *EBF1* and *EBF2* are negatively regulated by the exoribonuclease EIN5 in the presence of ET. The transcription factor EIN3 dimerizes and then activates the expression of target genes, including the transcription factor gene ethylene-response-factor1 (*ERF1*). ERF1, in turn, starts a transcriptional cascade of 100s of ET-regulated genes. The mechanism of ET signaling in plants is probably universal as all the elements identified in *Arabidopsis* are conserved in evolutionary distant plant species (Merchante et al., 2013).

## EIN3 PROTEIN CONTROL

Recent studies have highlighted the role of ubiquitin/proteasome pathway in various aspects of plant growth and development as the paradigm for plant hormone signaling. A ubiquitin/proteasome pathway has been demonstrated in auxin, gibberellin, abscisic acid and jasmonate signaling, and implicated in the salicylic acid, cytokinin, and brassinosteroid responses (Frugis and Chua, 2002; Vierstra, 2003; Smalle and Vierstra, 2004; Dreher and Callis, 2007). Three groups independently discovered that EIN3 is degraded by the 26S proteasome-dependent pathway, and that EBF1 and EBF2 are two proteins mediating EIN3 degradation (Guo and Ecker, 2003; Potuschak et al., 2003; Gagne et al., 2004). EBF1 functions constitutively by keeping EIN3 below a critical threshold, thereby repressing the ET response at low hormone concentrations. EBF2 acts mainly in silencing the signal by removing activated EIN3 so the plants can more rapidly resume normal growth (Binder et al., 2007). It has been shown, that ET can induce *EBF2* expression forming a negative feedback loop to desensitize ET signaling (Potuschak et al., 2003).

Moreover, EIN3 protein seems also to be quantitatively controlled by other signals. It has been found that glucose can promote EIN3 degradation by an unknown mechanism (Yanagisawa et al., 2003), whereas light can positively regulate EIN3 and EIL1 stability (Lee et al., 2006). Furthermore, two different phosphorylation sites, oppositely affecting the level of EIN3, have been identified, pointing out the involvement of the MAPK-dependent pathway in ET signaling (Yoo et al., 2008). In this model, MKK9 cascade phosphorylates EIN3 to promote its stability, whereas phosphorylation by an MAPK pathway mediated by kinase CTR1 promotes EIN3 degradation. An additional phosphorylation site, highly conserved in all members of the EIL family, was recently proved to be of fundamental importance for the dimerization of tomato EIL1 and crucial for its transcriptional activity (Li et al., 2012). As such, EIN3 may represent a central regulator of plant growth, capable of integrating various external, and internal signals. This is understandable, since most phytohormones are involved in multiple processes and influence each other through complex crosstalk strategies (Santner and Estelle, 2010). For many years the synergy or antagonism between ET and jasmonic acid (JA) signaling has been observed in many developmental and defense-related processes (Pauwels and Goossens, 2011). At least part of this crosstalk is mediated by the interaction of EIN3/EIL1 with JAZ proteins, which are repressors in jasmonate signaling (Kazan and Manners, 2012). JAZ proteins bind to EIN3/EIL1, thus suppressing the DNA-binding ability of EIN3. The emerging model, providing a plausible explanation for the synergy in many processes regulated by both hormones, emphasizes the role of ET in EIN3/EIL1 stabilization and jasmonate in EIN3/EIL1 release from the JAZ protein repression (Zhu et al., 2011). Another layer of the crosstalk between those two pathways is the interaction between the jasmonate-activated transcription factor MYC2 and EIN3 (Song et al., 2014). MYC2 interacts with EIN3 to attenuate ET-enhanced apical hook curvature, while EIN3 represses MYC2 to downregulate jasmonate-regulated plant defense against generalist herbivores (Zhang et al., 2014; **Figure 1**).

There is strong evidence regarding the involvement of ET in plant responses to nutritional stresses. Changed levels of ET production were reported as a result of nitrogen, phosphorus, potassium, calcium, and iron deficiency (Lynch and Brown, 1997; Benlloch-Gonzalez et al., 2010). A direct molecular link between ET signaling and iron metabolism was found (Lingam et al., 2011). EIN3/EIL1 can physically interact with FIT, a central regulator of iron acquisition in roots (**Figure 1**). Through this interaction, proteasomal degradation of FIT is reduced and leads to a higher level of expression of the iron acquisition genes. Another factor directly interacting with EIN3 is the Mediator complex subunit MED25 (Yang et al., 2014). Mediator is a conserved multisubunit complex, regulating the transcription by bridging transcription factors with RNA polymerase II. The MED25 subunit tunes up iron homeostasis but is also important for plant disease resistance, flowering, and organ size (Yang et al., 2014). ET is thereby one of the signals that triggers iron deficiency responses at the transcriptional and post-transcriptional levels. Recently, it was evidenced that ET is involved in the sulfur deficiency response with a special highlight on the role of small proteins of unknown function from the LSU/UP9 family (Moniuszko et al., 2013). It’s tempting to speculate that similar regulatory mechanisms influencing EIN3 stability and the wealth of interactions refer also to its close homologue, SLIM1.

## SLIM1 PROTEIN CONTROL

Not much is known about SLIM1 post-translational modifications or its protein partners. Reportedly, exogenous ET did not affect the expression of any of the *EIL* genes in *Arabidopsis*, tomato, tobacco, and mung bean (Chao et al., 1997; Tieman et al., 2001; Lee and Kim, 2003; Rieu et al., 2003) indicating the regulation at the post-transcriptional level. In contrast, transcriptional induction of *EILs* by ET or mechanical wounding was demonstrated in other plant species, such as petunia, carnation, banana, and rice (Waki et al., 2001; Shibuya et al., 2004; Iordachescu and Verlinden, 2005; Mbeguie et al., 2008; Hiraga et al., 2009). Interestingly, iron deficiency induces the expression of genes involved in ET synthesis and signaling, with *SLIM1* among them, in the *Arabidopsis* roots (Garcia et al., 2010). *SLIM1* is expressed predominantly in vascular tissues and despite the genetically evidenced importance of SLIM1 in sulfur response, its transcription level is not modulated by the changes of sulfur conditions (Maruyama-Nakashita et al., 2006). It is tempting to speculate that SLIM1 may require post-transcriptional mechanisms for the regulation of its performance. Presumably such regulation is accomplished by the highly selective ubiquitin/proteasome system removing SLIM1 protein while its function is not needed. However, unlike EIN3, whose protein level in the nucleus is affected by the ET and carbon status (Guo and Ecker, 2003; Potuschak et al., 2003; Yanagisawa et al., 2003), neither nuclear localization nor abundance of SLIM1 protein were changed by sulfur conditions (Maruyama-Nakashita et al., 2006). Interesting observations come from the recent studies of Aubry et al. (2014) revealing the crucial role of bundle sheath cells in sulfur metabolism. Despite the strong transcriptional upregulation of the whole sulfur assimilation pathway and the glucosinolates metabolism, the representation of *SLIM1* transcript was not increased, again pointing to the SLIM1 protein level control. It cannot be excluded, however, that other factors are controlling sulfur metabolism in this cell type. So far there is only one described protein partner of SLIM1 (**Figure 1**). MYB72, which is involved in induced systemic resistance, has been shown to interact physically with SLIM1 in the yeast two-hybrid assay (Van der Ent et al., 2008). Recognition of the beneficial microbes leads to the induction of *MYB72* and interaction of the protein with SLIM1 to trigger a jasmonate/ET-dependent resistance effective against a broad range of pathogens (Van der Ent et al., 2008). MYB72 together with MYB10 were recently found to be essential for plant survival under iron- deficiency, inducing the nicotianamine synthase gene *NAS4* necessary for proper metal homeostatsis (Palmer et al., 2013). Interestingly, MYB72 has also been described as a direct target of FIT, the root-specific central regulator of iron deficiency (Sivitz et al., 2012). This raises the question of whether the tandem MYB72–SLIM1 plays an additional regulatory role in sulfur deficiency responses. SLIM1, on the other hand, negatively regulates the expression of another MYB family member, ATR1/MYB34, thereby affecting glucosinolate biosynthesis in *Arabidopsis* roots (Maruyama-Nakashita et al., 2006). Another intriguing observation is that SLIM1, in contrast to the homodimers of EIN3, EIL1, and EIL2, exists in the monomeric form (Solano et al., 1998). It was suggested that the dimerization of EIL proteins is important in the stable binding to a pseudo-palindromic DNA sequence present in ET-responsive promoters, although it is still possible for monomeric proteins to bind to a shorter consensus (Yamasaki et al., 2005).

## SLIM1 BINDING TO DNA

Solano et al. (1998) have shown that the proteins from the *Arabidopsis* EIL family bind directly to primary ET response DNA elements, which are 28-nt imperfect palindromes found in the promoters of various ET-responsive genes. At the same time the 8-nt consensus binding sequence was defined for tobacco NtEIL1/TEIL, the transcription factor also believed to be involved in ET signaling (Kosugi and Ohashi, 2000). The similarity between those DNA regions is very high, however, the sequence essential for EIN3 binding was bound by TEIL with considerably less affinity than the TEIL binding sequence (*tebs*), showing differences in the binding preference between EIL family members (Kosugi and Ohashi, 2000). On the other hand, it was proved during *in vitro* studies that SLIM1 is able to bind to *tebs*, though the interaction is very unstable and only detectable with surface plasmon resonance but not by electro-mobility shift assay (Yamasaki et al., 2005). *Tebs* are present in the promoters of several sulfur deficiency-induced genes of *Arabidopsis*, the regulation of which is also controlled by SLIM1 (Maruyama-Nakashita et al., 2006). Additionally, the direct interaction of SLIM1 with 20-nt consensus, called the UPE-box was demonstrated (Lewandowska et al., 2010; Wawrzynska et al., 2010). The UPE-box contains two *tebs*, partially overlapping in opposite orientation to each other, and is only present in the promoters of several *Arabidopsis* genes strongly induced by sulfur deficiency (Wawrzynska et al., 2010). Among those genes, are genes encoding proteins from the LSU family, homologues of tobacco UP9C protein. Transgenic tobacco plants with lowered expression of *UP9C* showed the disturbed response of the ET signaling and synthesis pathways during conditions of sulfur deficiency, indicating a crosstalk between ET and sulfur metabolism in plants (Moniuszko et al., 2013). Interestingly, the UPE-boxes were also found in promoters of a co-regulated gene cluster induced by the cysteine precursor *O*-acetylserine, suggesting a potential function for SLIM1 in the sensing of sulfur status (Hubberten et al., 2012). A signaling function of *O*-acetylserine in sulfur assimilation by enteric bacteria had already been stated a long time ago (Ostrowski and Kredich, 1990); however, its role as a sensor of sulfur status in plants is still under debate.

## THE *slim1 Arabidopsis* MUTANTS CHARACTERISTICS

The *slim1* mutants are not able to induce expression of the high-affinity sulfate transporter SULTR1;2 and consequently sulfate uptake during sulfur deficiency. SLIM1 inactivation results in a 60% limitation of sulfate uptake rate and a 30% reduction in root length (in comparison to the wild-type plants). The metabolite analysis further suggests that *slim1* mutants may be experiencing the lowered supply of sulfate to the reduction pathway as evidenced by the significant decrease of glutathione content and overaccumulation of *O*-acetylserine in their shoots. Such metabolite profiles are characteristic for sulfur-deficient plants and caused by an insufficient sulfur amount for the cysteine synthesis pathway. Degradation of glucosinolates is another important aspect of sulfur limitation response. Glucosinolates are characteristic compounds for *Brassicales* participating in the defense against herbivores and pathogens (Halkier and Gershenzon, 2006). The *slim1* mutations concomitantly affect the expression of metabolic and regulatory genes of glucosinolate biosynthetic pathways. Consistent with the transcriptional changes, glucosinolates levels were shown to be higher in the *slim1* mutants, even under sulfur deficient conditions (Maruyama-Nakashita et al., 2006). These results provide strong evidence for the function of SLIM1 in the co-regulation of this sulfur recycling process in parallel with sulfate transport systems during sulfur deficiency. However, since the transcriptomic profile of the *slim1* mutants showed alterations in many, but not all, genes responsive to sulfate deficiency, one might expect other factors controlling these processes. Additionally, it was suggested recently that SLIM1 may possess a dual function as an activator at sulfur limitation and a repressor during normal sulfur status (Matthewman et al., 2012). This was evidenced by higher sulfate uptake by the *slim1* mutants on a normal sulfate supply which was consistent with the higher transcript level for *SULTR1;1* (Maruyama-Nakashita et al., 2006).

## SLIM1 INTERPLAY WITH microRNA395

Another level of control in gene expression is the regulation by microRNAs (miRNAs), which are a class of naturally occurring, small non-coding RNA molecules. They are partially complementary to one or more mRNA molecules, and their main function is to affect the stability of these molecules in a variety of manners, including translational repression, mRNA cleavage, and deadenylation (Voinnet, 2009). Functionally, miRNAs are involved in a variety of developmental processes in plants, including stress responses with nutrient deficiencies. Among those, miR395 in *Arabidopsis* was identified as being involved in the regulation of sulfate transport and assimilation targeting the mRNAs of three isoforms of ATP sulfurylase and one transporter SULTR2;1 facilitating inter-organ transport of sulfate (Bonnet et al., 2004; Jones-Rhoades and Bartel, 2004). The expression of miR395 is drastically upregulated under sulfur limitation and its induction is directly or indirectly controlled by SLIM1 (Kawashima et al., 2009; **Figure 1**). The cell-type specific expression pattern between miR395 and its target transcripts enables the fine-tuning of the sulfur assimilation rate (Kawashima et al., 2011). Especially interesting is the unexpected positive correlation of expression between miR395 and targeted *SULTR2;1* during sulfur deficiency. It enables restriction of the SULTR2;1 transporter to xylem parenchyma cells, thus together with sulfate reduction shut off, providing for efficient translocation of sulfate from roots to shoots (Kawashima et al., 2009). Additionally, grafting experiments provided convincing evidence that miR395 are phloem-mobile, suggesting their role as long-distance signaling molecules, and underlying the importance of systemic regulation of plant response to varying sulfur levels (Buhtz et al., 2010). Recently, it was demonstrated that the trigger of miR395 accumulation is linked rather to internal sulfate levels and not external sulfate availability (Matthewman et al., 2012), again pointing out to the *O*-acetylserine as an activating signal and to the involvement of SLIM1. Moreover, it was also shown that the redox signaling plays an important role in miR395 induction during sulfur deficiency, placing SLIM1 downstream in the regulatory cascade (Jagadeeswaran et al., 2014). However, whether SLIM1 itself is a target of redox signaling has not yet been determined.

Interestingly, EIN3 also participates in the control of miRNA by integrating different developmental and environmental cues and directly binding to the promoters of miR164 activating leaf senescence processes (Li et al., 2013).

## CONCLUSION AND FUTURE PROSPECTS

Due to the sessile life cycle, plants have developed different strategies to adapt to adverse environmental stresses. Plant growth and development is largely impaired by nutrient deficiencies; therefore to maintain good productivity in plant breeding, it is essential to understand fully those mechanisms. In this review, we focused on SLIM1 as the only described transcriptional regulator dedicated to plant response to sulfur deficiency. It belongs to the same protein family of transcription factors as EIN3. Contrary to SLIM1, regulation of EIN3 stability, interaction with other proteins as well as the whole signaling pathway leading to transcriptional response is already well described (**Figure 1**). It is of interest as to whether the same level of complexity can be expected in sulfur deficiency signaling. Still, the exact signaling cascade leading from sensing to activating the expression of the SLIM1-dependent gene set, resulting in sulfur metabolism reprogramming, needs to be clarified and future studies are required to reveal the molecular components, with a special emphasis on the role of *O*-acetylserine (**Figure 1**). Such studies should also concentrate on the investigation of post-transcriptional modifications of SLIM1 influencing its functionality under different sulfur regimes, as well as its direct interaction with specific DNA sequences. We must be cautions, however, in drawing general conclusions and remember that SLIM1 is present mostly in the vascular tissues thus its action might be predominantly connected with the translocation of sulfate between plant parts rather than governing the whole plant sulfur metabolism.

## AUTHOR CONTRIBUTIONS

Anna Wawrzyńska drafted the manuscript and prepared the figure; Agnieszka Sirko revised it critically for important intellectual content.

## Conflict of Interest Statement

The authors declare that the research was conducted in the absence of any commercial or financial relationships that could be construed as a potential conflict of interest.
